# Left hand polydactyly: a case report

**DOI:** 10.1186/1757-1626-1-346

**Published:** 2008-11-24

**Authors:** Nicola Mumoli, Daniele Gandini, Edris Kalanzi Wamala, Marco Cei

**Affiliations:** 1 Section of Emergency Medicine, Department of Internal Medicine, Ospedale Civile Livorno, 57100 Livorno, Italy; 2 Department of Plastic Surgery, Ospedale Cisanello Pisa, 56124 Pisa, Italy

## Abstract

**Background:**

Polydactyly is a congenital anomaly with a wide range of manifestations that occurs in many forms, ranging from varying degrees of mere splitting to completely duplicated thumb. When duplication occurs alone, it is usually unilateral and sporadic.

**Case presentation:**

In this case report we describe an otherwise healthy 19-year-old woman of Tibetan heritage with isolated left hand preaxial polydactyly. She experienced working related difficulties in her daily yak's milking. She subsequently underwent surgical correction, and the over number thumb was removed with associated meticulous skeletal and soft tissue reconstruction.

**Conclusion:**

Polydactyly is the most common congenital digital anomaly of the hand and foot. It can occur in isolation or as part of a syndrome. Surgery is necessary to create a single, functioning thumb and is indicated to improve cosmesis. Skin, nail, bone, ligament, and musculoskeletal elements must be combined to reconstruct an optimal digit. In this case (Tibetan society is almost exclusively a sheep-breeding one) surgery was necessary to leave a single, functioning thumb for her work as yak milkmaid.

## Background

Polydactyly of the hands or feet is a common birth deformity that occurs in many forms, ranging from varying degrees of mere splitting to completely duplicated thumb. Preaxial polydactyly is the most common of congenital hand anomalies. It can occur in isolation or as part of a syndrome. Isolated polydactyly is often autosomal dominant, while syndromic polydactyly is commonly autosomal recessive [1,2]. Polydactyly is classified into preaxial, central, and postaxial types. Preaxial polydactyly, the most common type, refers to the duplication of the first digital ray [3].

Several classfications were proposed, among which Wassel's classification [4] (table 1) is being widely used in clinical fields. Interestingly sometimes clinically recognized types do not correspond with the findings recorded at surgery. Reason behind it is the presence of cartilaginous epiphysis which does not show bifurcation level between two duplicated components in immature hand. So the classifications become inappropriate to some extent. It has been understood that radiological classification alone has little value in detection of clinical types as well as in preoperative assessment.

**Table 1:** Classification: Types I to VII based on level of duplications

- I: bifid distal phalanx
- II: duplicated distal phalanx

- III: bifid proximal phalanx

- IV: duplication of proximal phalanx which rest on broad metacarpal

- V: bifid metacarpal

- VI: duplicated metacarpal

- VII: triphalangism

## Case presentation

**Figure 1:**
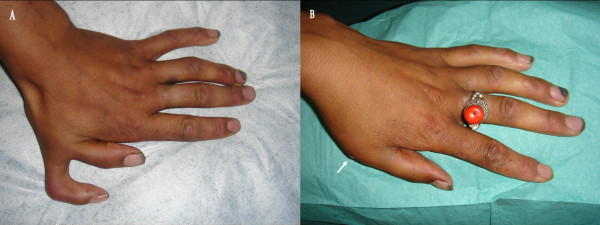
**A: the figure showed a left preaxial hexadactyly with thumb duplication**. B: the figure showed the suture (arrow) after removing the duplicated thumb.

A healthy 19-year-old Tibetan woman was admitted because of isolated left hand preaxial polydactyly (figure 1A). She experienced working related difficulties in her daily yak's milking. Physical examination revealed preaxial hexadactyly with thumb duplication. Extra digit was mild hypoplasic, had normal sensation, but the patient was unable to move it independently.

Radiographs showed extra thumb containing two proximal phalanges (type IV polydactyly, according to the Wassel's classification) (table 1).

The patient subsequently underwent surgical correction, and the duplicated thumb was removed with associated meticulous skeletal regularisation and soft tissue closure (figure 1B).

Healing was uneventful, and the patient was discharged with no occurrence of surgery related inconvenient.

## Discussion

Polydactyly is the most common congenital digital anomaly of the hand and foot. It can occur in isolation or as part of a syndrome. Isolated polydactyly is often autosomal dominant, while syndromic polydactyly is commonly autosomal recessive [1,2]. Polydactyly is classified into preaxial, central, and postaxial types. Preaxial polydactyly, the most common type, refers to the duplication of the first digital ray [3].

Radiographs of the affected limb are recommended to show whether the rudimentary digit contains skeletal elements. The degree of deviation of the digit and the size of the articulating metacarpal or metatarsal also may be helpful in surgical planning [5].

Surgery is necessary to create a single, functioning thumb. Typically this is performed around one year of age, before the development of pinch and fine motor function [6]. As mentioned below, simply removing one of the two thumbs will not suffice, as each of the split thumbs has elements that need to be combined to recreate the best possible thumb. As a result, surgical treatment usually entails removing the bony elements of the smaller thumb and reconstructing the remaining skin, tendons, ligaments, joints, and fingernails to form a new thumb. Even in the best of situations, the resulting thumb may smaller than the child's other, normal thumb. Furthermore, given the possibility of recurrent deformity in a small percentage of patients, additional operations may be needed later in life. It is important to evaluate and treat the skin, nail, bone, and the ligaments in a simultaneous manner in order to obtain a good reconstruction and to decrease both the complications and the need for subsequent operations [7,8].

## Conclusion

Polydactyly is the most common congenital digital anomaly of the hand and foot. The frequency of polydactyly varies widely among populations. It may be an isolated condition or part of a congenital syndrome. Polydactyly is generally classified into three major groups: medial ray (preaxial), central ray and lateral ray (postaxial).

Because hand and foot polydactyly are associated with congenital defects in 23.4% of patients, genetic workup and thorough medical examination in these patients is recommended [8].

Until a decade ago, the standard recommendation was simple excision of one of the duplicates. Problems of deformity, instability, and weakness became apparent, and reconstructive options were developed. With current techniques, good results are usual, although secondary reconstructive procedures may be required. Simple excision is seldom indicated. Combination procedures, involving core tissues of bone, joint, and tendon or peripheral tissue (neurovascular, subcutaneous and skin) or sometimes all of these, are preferred for reconstruction. The major current problem is to achieve the maximal good result with a minimal number of surgical operations. Careful clinical and radiographic evaluation should be made prior to treatment to achieve good functional and cosmetic results. Most cases are treated during childhood before walking age. Adult cases are more rare, and surgical management of the deformity is still debated. Nevertheless, surgery can be performed at any age with good results.

The psychological burden of having an extra finger has been linked with psychosis in adulthood and the deformity is cosmetically unacceptable in many cultures. Polydactyly causes social embarrassment and often results in compromised to move the hand independently. Furthermore, it often has a severe impact on the patients self esteem and jeopardizes his or her quality of life. The treatment plan is formulated and tailored specifically for each individual patient. Thorough evaluation and diagnosis are among most important aspects of overall patient's management. The evaluation and treatment plan is based on the outcome of analysis of the patient's medical history, social history, motivation, social and psychological disturbances. A patient's ultimate satisfaction with treatment outcome often depends upon attention to the patient's chief concerns. Patients with unrealistic expectations must be counselled, so that they understand treatment limitations and likely outcomes before initiating surgical therapy. Co-operative studies between surgeons and psychiatrists have provided some valuable guidelines in the evaluation and selection of patients for corrective surgery.

In this case (Tibetan society is almost exclusively a sheep-breeding one) surgery was necessary to leave a single, functioning thumb for her work as yak milkmaid.

## Competing interests

The authors declare that they have no competing interests.

## Authors' contributions

NM, DG, EKW, MC, analyzed and interpreted the patient data and all gave a major contributor in writing the manuscript. All authors read and approved the final manuscript.

## Consent

Written informed consent was obtained from the patient for publication of this case report and accompanying images. A copy of the written consent is available for review by the Editor-in-Chief of this journal.
